# Development and Optimization of a Redox Enzyme-Based Fluorescence Biosensor for the Identification of MsrB1 Inhibitors

**DOI:** 10.3390/antiox13111348

**Published:** 2024-11-02

**Authors:** Hyun Bo Shim, Hyunjeong Lee, Hwa Yeon Cho, Young Ho Jo, Lionel Tarrago, Hyunggee Kim, Vadim N. Gladyshev, Byung Cheon Lee

**Affiliations:** 1 College of Life Sciences and Biotechnology, Korea University, Seoul 02841, Republic of Korea; simhb0708@korea.ac.kr (H.B.S.); hjl2674@korea.ac.kr (H.L.); chway4418@korea.ac.kr (H.Y.C.); jast1@naver.com (Y.H.J.); hg-kim@korea.ac.kr (H.K.); 2 College of Engineering, Institute of Green Manufacturing Research Center, Korea University, Seoul 02841, Republic of Korea; 3 GERONMED, Co., Ltd., Hoegi-ro 117-3, Seoulbiohub, Research Building, 5F, 504, Seoul 02455, Republic of Korea; 4 French National Institute for Agriculture, Food, and Environment (INRAE), Aix Marseille University, Biodiversité et Biotechnologie Fongiques (BBF), 13385 Marseille, France; lionel.tarrago@inrae.fr; 5 Brigham and Women’s Hospital, Harvard Medical School, Boston, MA 02115, USA; vgladyshev@rics.bwh.harvard.edu

**Keywords:** methionine sulfoxide reductase B1, redox protein-based fluorescence biosensor, high-throughput screening, inhibitor, inflammation, circularly permuted yellow fluorescence protein

## Abstract

MsrB1 is a thiol-dependent enzyme that reduces protein methionine-*R*-sulfoxide and regulates inflammatory response in macrophages. Therefore, MsrB1 could be a promising therapeutic target for the control of inflammation. To identify MsrB1 inhibitors, we construct a redox protein-based fluorescence biosensor composed of MsrB1, a circularly permutated fluorescent protein, and the thioredoxin1 in a single polypeptide chain. This protein-based biosensor, named RIYsense, efficiently measures protein methionine sulfoxide reduction by ratiometric fluorescence increase. We used it for high-throughput screening of potential MsrB1 inhibitors among 6868 compounds. A total of 192 compounds were selected based on their ability to reduce relative fluorescence intensity by more than 50% compared to the control. Then, we used molecular docking simulations of the compound on MsrB1, affinity assays, and MsrB1 activity measurement to identify compounds with reliable and strong inhibitory effects. Two compounds were selected as MsrB1 inhibitors: 4-[5-(4-ethylphenyl)-3-(4-hydroxyphenyl)-3,4-dihydropyrazol-2-yl]benzenesulfonamide and 6-chloro-10-(4-ethylphenyl)pyrimido[4,5-b]quinoline-2,4-dione. They are heterocyclic, polyaromatic compounds with a substituted phenyl moiety interacting with the MsrB1 active site, as revealed by docking simulation. These compounds were found to decrease the expression of anti-inflammatory cytokines such as *IL-10* and *IL-1rn*, leading to auricular skin swelling and increased thickness in an ear edema model, effectively mimicking the effects observed in MsrB1 knockout mice. In summary, using a novel redox protein-based fluorescence biosensor, we identified potential MsrB1 inhibitors that can regulate the inflammatory response, particularly by influencing the expression of anti-inflammatory cytokines. These compounds are promising tools for understanding MsrB1’s role during inflammation and eventually controlling inflammation in therapeutic approaches.

## 1. Introduction

Methionine is a versatile amino acid that plays a crucial role in various physiological processes, such as protein synthesis, methylation, antioxidant activity, detoxification, or immune function [1,2,3,4,5]. Methionine can be easily oxidized by reactive oxygen species (ROS) to methionine sulfoxide (MetO), which exist as *R*- and *S*-diastereomers (Met-*R*-O and Met-*S*-O, respectively). Such modification can alter the protein’s function and potentially lead to metabolic dysfunction or the onset of disease [6,7,8,9,10,11,12,13,14,15].

In mammals, methionine sulfoxide reductase B1 (MsrB1) is a selenoprotein located in cytosol and nucleus that reduces Met-*R*-O back to methionine in protein. Due to its catalytic activity, MsrB1 is recognized not only for its antioxidant effects but also as an enzyme that repairs damaged proteins by using various oxidized proteins as substrates [16,17]. Furthermore, many studies indicate that MsrB1 is involved in the functional regulation of substrate proteins, such as the calmodulin kinase II, β-amyloid peptide, actin, and others, in conjunction with ROS or methionine-oxidizing enzymes, acting similarly to a reversible post-translational modification [18,19,20,21,22]. For example, molecules interacting with CasL (MICAL) oxidize two conserved methionine residues in actin, leading to actin depolymerization. This oxidized actin can then be reduced by MsrB1, allowing it to repolymerize and contribute to the regulation of the immune response in macrophages. Along with this association, deletion of MsrB1 caused suppression of anti-inflammatory cytokine expression, slightly enhancing proinflammatory cytokine expression when it was subjected to LPS stimulation [19,20]. Given that MsrB1 regulates biological functions via redox state changes in methionine in certain proteins, the underlying mechanism of regulatory processes, signaling pathways, and mechanisms connections to disease development are not yet well understood. Efforts have been made to identify inhibitors and activators of MsrA and MsrB, as well as to develop high-throughput screening systems to facilitate this process [23,24]. Through these efforts, analogs of fusaricidin A have been identified as activators of both MsrA and MsrB. However, there is still a need for more inhibitors, particularly for MsrB1, given the potential therapeutic benefits of enhancing inflammation in certain cases, such as chronic infections, vaccine adjuvants, cancer immunotherapy, and immunocompromised patients [25,26,27,28,29]. While inflammation-enhancing drugs are less common than anti-inflammatory drugs, they play crucial roles in medical contexts where boosting the immune response is advantageous. Therefore, the discovery of inhibitors capable of modulating MsrB1 activity is urgently needed for pharmacological applications and to advance research in this area.

The advent of redox protein-based fluorescence biosensors has greatly enhanced our ability to detect various oxidation states in biological systems. Following the principle demonstrated by Hyper, which senses hydrogen peroxide through structural changes in circularly permuted yellow fluorescent protein (cpYFP) in response to reactive oxygen species interacting with oxidative stress regulator (OxyR) [30], numerous redox protein-based fluorescence biosensors have been developed. These include MetSOx, which detects protein and free Met-*S*-O; MetROx, reacting with protein-bound Met-*R*-O; TYfR, which targets free Met-*R*-O; and tpMetROG, which targets Met-*R*-O in a specific protein [31,32,33]. These biosensors typically rely on enzyme-based substrate binding, followed by structural changes in cpYFP due to disulfide bond exchange.

Building on this principle, we developed a novel protein-based fluorescence biosensor utilizing MsrB1 for the quantitative measurement of Met-*R*-O. This biosensor was employed in high-throughput screening to identify potential inhibitors of MsrB1. The inhibitor compounds were analyzed using molecular docking simulations to provide theoretical insights, followed by experimental validation through NADPH consumption assays, MST binding assays, and HPLC analysis. Among the tested compounds, two exhibited superior inhibitory effects compared to others and were further demonstrated to regulate inflammatory responses effectively.

## 2. Materials and Methods

### 2.1. Cloning and Purification of Recombinant Proteins

The coding sequences of mouse MsrB1 and human thioredoxin1 (Trx1) were synthesized (Cosmogenetech, Seoul, Republic of Korea) and subsequently amplified by PCR. Site-directed mutagenesis of human Trx1 (cysteine393 to serine393) and mouse MsrB1 (active form: selenocysteine95 to cysteine95, inactive form: selenocysteine95 to serine95) was performed using EZchange™ Site-directed Mutagenesis kit (Enzynomics, Daejeon, Republic of Korea). cpYFP from the HyPer sensor [30] was cloned and used to construct recombinant DNA, sequentially assembling MsrB1/cpYFP/Trx1 in a pET-28a vector named RIYsense (Addgene, Watertown, MA, USA). A small-scale solubility test was conducted to determine the optimal conditions for protein expression and solubility in BL21, Rosetta, and Rosetta2 pLysS (Novagen, Madison, WI, USA). Transformed cells were cultured in 5 mL in Luria–Bertani medium (Formedium, Norfolk, UK) and grown overnight in the shaking incubator at 37 °C. Next, cells were harvested and lysed using BugBuster protein extraction reagent (Novagen, Madison, WI, USA). The cell lysates were analyzed by SDS-PAGE and used to determine optimal protein expression with Rosetta2 (DE3) pLysS strain.

The recombinant RIYsense construct was transformed into Rosetta2 pLysS cells and cultured in an LB medium containing ampicillin. When the optical density (O.D.) at 600 nm reached 0.6~0.8, protein expression was induced by adding 0.7 mM Isopropyl β-D-1-thiogalactopyranoside (IPTG) (Gold Biotechnology, St Louis, MO, USA) at 18 °C for 18 h. Cells were harvested by centrifugation at 3500 rpm and then resuspended in a buffer containing 20 mM Tris(hydroxymethyl)aminomethane (Tris) (Daejung, Gyeonggi-do, Republic of Korea), 150 mM NaCl (Daejung, Republic of Korea), and 5 mM β-mercaptoethanol (Daejung, Republic of Korea) at pH 8.0. The resuspended cells were lysed by sonication and subjected to centrifugation at 13,000 rpm for 60 min. The supernatant was filtered using a 0.45 µM cellulose acetate syringe filter (Advantec, Tokyo, Japan) and purified using affinity chromatography. The sample was loaded onto a HisTrap HP column (GE Healthcare, Seoul, Republic of Korea) and eluted with buffer containing 20 mM Tris-HCl, 150 mM NaCl, 5 mM β-mercaptoethanol, and 500 mM imidazole at pH 8.0. The purified protein was concentrated using 50 mL Amicon Ultra centrifugal filters with 30-kDa cutoffs and stored at −80 °C.

### 2.2. Fluorescence Spectroscopic Characterization of RIYsense Biosensor Protein

The purified RIYsense protein was reduced by using 50 mM Dithiothreitol (DTT) for 30 min at room temperature (RT). After reduction, the protein was desalted using a HiTrap desalting column (GE Healthcare, Seoul, Republic of Korea) with 20 mM Tris-HCl at pH 8.0 and diluted to a final concentration of 4 μM for further use. Fluorescence measurements were conducted using a TECAN SPARK multimode microplate reader (Molecular Devices, San Jose, CA, USA) with samples placed in a 96-well black microplate. RIYsense protein (100 μL), active form or inactive form, was incubated with or without 10 μL of 500 μM N-AcMetO in 20 mM Tris-HCl buffer (pH 8.0) for 10 min at RT, and the absorbance spectrum was recorded from 380 nm to 700 nm. Then, the emission spectrum was measured from 500 nm to 600 nm with excitation at 420 nm, while the excitation spectrum was measured from 380 nm to 500 nm with emission at 545 nm. The two peaks at 420 nm and 485 nm in the excitation spectrum with emission at 545 nm were used to calculate the quantity of protein methionine sulfoxide (MetO) by using the ratio of fluorescence intensities (RFI = 485 nm/420 nm).

For functional analysis, 100 μL of RIYsense protein in 20 mM Tris-HCl buffer (pH 8.0) was incubated with 10 μL of 500 μM N-acetylmethionine sulfoxide (N-AcMetO) or 10 μL of 500 μM N-acetylmethionine (N-AcMet) for 10 min at RT. After incubation, the RFI value was calculated following fluorescence spectroscopic analysis. For kinetic analysis, 100 μL of RIYsense protein was incubated with 10 μL of 0, 120, 250, or 500 μM N-AcMetO for 900 s at RT. The RFI value was measured every 30 s for the entire 900 s period. The RFI value at each time point was then divided by the RFI value at 0 s, and the result was expressed as R/R0.

### 2.3. RIYsense Biosensor Protein Incubation at Various PH

The active and inactive forms of RIYsense were incubated with N-AcMetO (oxidized) or without N-AcMetO (reduced) at various pH levels using different buffers: 50 mM Sodium phosphate (pH 5.8, 6.5); 20 mM Hepes (pH 7.0, 7.5); and 20 mM Tris-HCl (pH 8.0, 8.5, 9.0). Prior to the assay, the sensor was pre-treated with 50 mM DTT for 30 min to ensure reduction, then desalted using a HiTrap desalting column (GE Healthcare) with each respective buffer.

For the assay, the reduced sensor (4–6 μM) was incubated with 10 mM N-AcMetO in a black microplate well (final volume of 100 μL) for 20 min at 25 °C. This was performed for each buffer responsible for the specific pH. To measure the maximum dynamic range of the sensor, the fluorescence ratio was calculated by dividing the fluorescence at 485 nm excitation by the fluorescence at 420 nm. The pH range that produced the highest sensor response was determined by calculating the ratio of the oxidized to the reduced RIYsense at each pH level.

### 2.4. High-Throughput Screening to Identify MsrB1 Inhibitor Using RIYsense Protein

A high-throughput screening of chemical compounds was performed to identify inhibitors of MsrB1. A total of 6868 compounds, provided by the Korea Chemical Bank, were utilized for the screening. The RIYsense protein was first incubated with 50 mM DTT for 30 min, followed by desalting and dilution to a concentration of 4–6 μM using 20 mM Tris-HCl at pH 8.0. Each well of a 96-well black microplate (SPL Life Science, Gyeonggi-do, Republic of Korea) was filled with 100 μL of the diluted RIYsense protein solution, with the inactive form of RIYsense used as a control. Subsequently, 1 μL of each chemical compound dissolved in dimethylsulfoxide (DMSO) was added to the respective wells, and the plate was incubated for 10 min. After incubation, 10 μL of 500 μM N-AcMetO serving as the substrate for the sensor was added to each well and incubated for 10 min at RT. The RFI value from RIYsense incubated with 500 μM N-AcMetO without any chemical compounds was considered 100% when calculating the percentage of relative RFI. This baseline was used to compare the effects of different chemical compounds on the RIYsense protein’s activity.

### 2.5. Molecular Docking Simulations of MsrB1 Inhibitors Using Autodock Vina Software

The 3D structure of mouse MsrB1 was obtained from the Protein Data Bank (PDB) website. Ligand–protein docking was performed to predict the optimal position of ligands within the active site of the receptor to identify conformation with the lowest binding energy. Based on HTS results conducted using the characteristics of the B1YT sensor, AutoDock Vina was selected as the virtual screening program to find binding free energy value and RMSD (Root Mean Square Deviation). We also used AutoDock Vina to remove small molecules from the structure. A total of 246 SMILES files consisting of 192 inhibitor candidates and 52 activator candidates were provided by Korea Compound Bank. All SMILES files were converted to SDF format using OBABEL, and the MMFF94 (Merck Molecular Force Field 94) was applied to each ligand. Subsequently, the structures were converted to pdbqt format using AutoDockTools 1.5.6 software, with the addition of polar hydrogens and Kollman charges. The grid box is centered on the active site of MsrB1, catalytic selenocysteine (U95), and resolving cysteine (Cys41). Grid box sizes were optimized to ensure coverage of the active site without being excessively large. The grid box was set to cover the active site of the crystal structure with the following dimension in Å: center (X, Y, Z) = (7.17, 3.04, 32.17) and four different grid sizes as 18 Å × 18 Å × 18 Å, two bigger sizes as 32 Å × 32 Å × 32 Å, 40 Å × 40 Å × 40 Å, and 60 Å × 60 Å × 60 Å, with the largest grid encompassing the entire receptor. Docking parameters for AutoDock Vina were set number modes at 10, energy range of 4, and exhaustiveness at 10. Binding free energy scores (more negative values indicating stronger binding) and RMSD values of ≤2 Å were used to discover inhibitor candidates.

### 2.6. Microscale Thermophoresis (MST) Binding Assay for MsrB1 Inhibitors

MST analysis was performed using the Monolith NT.115 system (NanoTemper Technologies, München, Germany). Recombinant mouse MsrB1 protein was labeled with the Monolith His-Tag Labeling Kit RED-tris-NTA 2nd Generation (NanoTemper Technologies, Germany). The protein was prepared at a concentration of 200 nM, and the RED-tris-NTA 2nd Generation dye was diluted to 100 nM in the PBS-T (Tween 20) buffer. A mixture of 100 μL of 200 nM protein and 100 μL of 100 nM dye was incubated at RT for 30 min. After incubation, the sample was centrifuged at 13,000 rpm at 4 °C for 10 min, and the supernatant was transferred to a new tube, completing the protein labeling process and preparing the sample for analysis. Inhibitor compounds were serially diluted in DMSO, starting at a concentration of 1.25 mM (1:1 dilution, 16 points), and then each of the 16 dilutions was further diluted fivefold with PBS-T buffer. Next, 10 μL of the diluted compounds were mixed with 10 μL of the labeled protein, followed by resuspension and incubation for 10 min at RT. The samples were loaded into capillaries in serial order and analyzed using the Monolith NT.115 system at 60% LED power and 60% MST power. Changes in normalized fluorescence, dependent on the concentration of the compounds after 30 s of MST on time, were analyzed to determine Kd values.

### 2.7. Activity Assay of MsrB1 by NADPH Consumption and HPLC

The activity of MsrB1 was assessed over a 900-s period in a 20 mM 4-(2-hydroxyethyl)-1-piperazineethanesulfonic acid (HEPES) buffer at pH 7.5 and 37 °C. The reaction mixture contained 500 μM N-AcMetO, 6 μM MsrB1, 2 μM mouse thioredoxin, 1 μM mouse thioredoxin reductase, 50 μM NADPH, and approximately 50 μM of a chemical compound. All components, except for the chemical compound, substrate (N-AcMetO), and NADPH, were pre-mixed. The chemical compound was added and incubated for 10 min. NADPH (50 μM) and 500 μM N-AcMetO were added before absorbance measurements began. The rate of NADPH oxidation was monitored by measuring absorbance at 340 nm every 30 s until the reaction was complete.

Methionine sulfoxide reductase B1 activity was determined using dabsylated MetO as substrate. The reaction mixture (100 μL) contained 20 mM HEPES (pH 7.5), 40 mM DTT, 400 μM dabsyl-MetO, 1~2 μg of purified protein, and 1% DMSO or 100 μM inhibitors. The reaction was conducted for 30 min at 37 °C in a water bath and stopped by adding 200 μL of acetonitrile and vortexing. The reaction product, dabsyl-Met, was analyzed by HPLC using a ZORBAX RX-C18 column (5 μm; 4.6 × 150 mm). The column was pre-equilibrated with 0.14 M sodium acetate (pH 6.1), 0.5 mL/L triethylamine, and 30% acetonitrile. A total of 50 μL of the sample was loaded onto the column. The flow rate was 1 mL·min^−1^, and a linear gradient elution mode was used (30% to 70%). Dabsyl-Met was detected via absorbance at 436 nm.

### 2.8. Analysis of Cytokine Expression in Macrophage

Bone marrow-derived macrophages (BMDMs) were generated by culturing bone marrow cells isolated from 8-week-old male C57BL/6 mice and MsrB1 knockout (KO) mice. The cells were cultured in high-glucose Dulbecco’s Modified Eagle Medium (DMEM) (Cytiva, Marlborough, MA, USA) supplemented with 10% fetal bovine serum (FBS), 100 U·mL^−1^ penicillin-streptomycin (Gibco, Waltham, MA, USA), 20 mM HEPES (Gibco, Waltham, MA, USA), and 50 ng·mL^−1^ recombinant mouse macrophage colony-stimulating factor (M-CSF) (BioLegend, San Diego, CA, USA). On day 3, 10 mL of fresh medium containing 50 ng·mL^−1^ M-CSF was added to the cultures. On day 5, cells were treated with 1 μM MsrB1 inhibitors. On day 6, cells were treated with LPS (BioLegend, San Diego, CA, USA) for 24 h. Following treatment, cells were harvested, and total RNA was extracted using the High-Capacity cDNA Reverse Transcription Kit (Applied Biosystems, Foster City, CA, USA). Gene expression of *IL-10* and *IL-1rn* mRNA was analyzed using the QuantStudio 3 Real-Time PCR system [34].

### 2.9. 12-O-Tetradecanoylphorbol-13-Acetate (TPA) Induced Acute Skin Inflammation Model

Six-week-old male wild-type (WT) C57BL/6 mice and MsrB1 knockout (KO) mice were used in this experiment. The mice were provided with 50 mL water containing either 0.5 μM MsrB1 inhibitors dissolved in 1% DMSO or 1% DMSO alone (control) for 21 days. After this period, 50 μg·mL^−1^ of TPA was applied to the right ear of each mouse, while the left ear received acetone as a control. Twenty hours after the initial TPA application, a second dose was administered. The mice were then sacrificed, and ear tissue samples were collected. The tissues were fixed in 10% formalin, dehydrated, embedded in paraffin, and sectioned into slices 3–5 μm thick. The sections were deparaffinized, rehydrated, and stained with hematoxylin and eosin (H&E) for histological examination under microscopy.

### 2.10. Statistical Analysis

Data values are expressed as mean ± standard deviation. *p*-values were obtained with the unpaired, two-tailed Student’s *t*-test.

## 3. Results

### 3.1. Development of a Protein-Based Fluorescence Biosensor Using MsrB1 for Detecting Methionine-R-Sulfoxide

**Figure 1:**
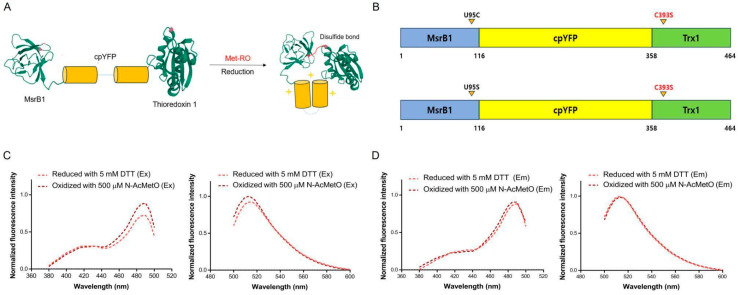
Characteristics of RIYsense biosensor. (**A**) Diagram of RIYsense protein and its potential mechanism of action. (**B**) Schematic view of the primary structure of the active and inactive form of RIYsense. Excitation and emission spectra of reduced and oxidized RIYsense in (**C**) the active form and (**D**) the inactive form.

Redox enzymes, in combination with circularly permuted fluorescent proteins, have been developed to represent a powerful approach to the development of protein-based fluorescence biosensors. We generate the biosensor protein named RIYsense, which uses the redox enzyme MsrB1 in conjunction with circularly Fpermuted yellow fluorescent proteins (cpYFP) for detecting methionine-*R*-sulfoxide in protein. This RIYsense is a recombinant protein that is composed of mouse MsrB1, where the catalytic selenocysteine, U95, is replaced with cysteine, cpYFP, and human Trx1, where the resolving cysteine, Cys393, is replaced with serine to stably maintain the inter-disulfide bond between MsrB1 and Trx1 (Figure 1A). To characterize the spectrophotometric function of this biosensor protein, we prepared both active and inactive forms of the recombinant RIYsense protein. The active form contains a catalytic cysteine in the MsrB1 domain, while the inactive form has the catalytic cysteine replaced by serine (Figure 1B). Both forms of the biosensor protein were reduced using dithiothreitol (DTT) and then incubated with N-AcMetO, a substrate that mimics the protein methionine sulfoxide (MetO). The data were presented as normalized fluorescence intensity (NFI) of the excitation spectrum, with all values divided by the maximum value of the excitation spectrum. The result showed a single peak at 520 nm in the emission spectrum and two peaks at 420 nm and 485 nm in the excitation spectrum for both the active and inactive forms. When the active form was incubated with N-AcMetO, the NFI value increased at 485 nm in the excitation spectrum and 520 nm in the emission spectrum, while the inactive form showed no change (Figure 1C,D). Additionally, the NFI value at 420 nm remained unchanged when the active form was incubated with N-AcMetO. This indicates that the biosensor protein can be used to quantify protein methionine sulfoxide by using the ratio of fluorescence intensities (RFI) at the two peaks in the excitation spectrum (RFI = 485 nm/420 nm). Therefore, this demonstrates that RIYsense is effective for detecting protein methionine sulfoxide, particularly for methionine-R-sulfoxide, which is the substrate of MsrB1.

### 3.2. RIYsense Biosensor Protein Characterization According to the Substrate N-AcMetO

**Figure 2:**
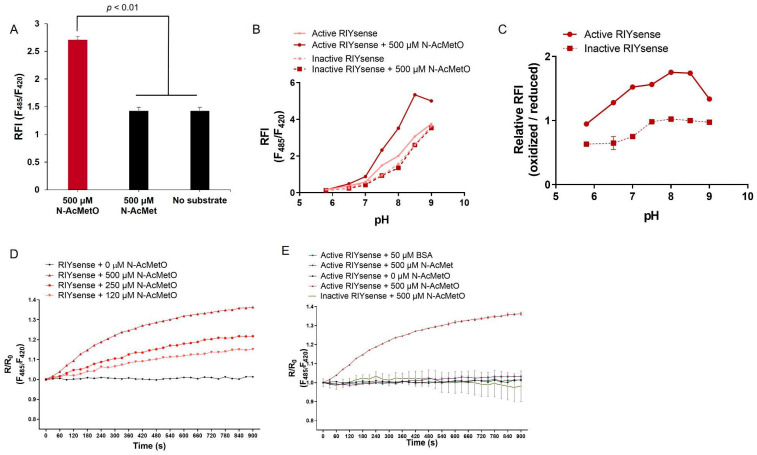
Substrate specificity of RIYsense. (**A**) RFI of RIYsense incubated with N-AcMetO or N-AcMet. (**B**) The active and inactive forms of RIYsense were incubated with N-AcMetO (oxidized) or without N-AcMetO (reduced) at different pH levels, and (**C**) the relative RFI was presented as the ratio of oxidized to reduced forms of both active and inactive RIYsense across various pH levels. (**D**) Kinetic analysis of RIYsense incubated with various concentrations of N-AcMetO. (**E**) Kinetic analysis of the active form of RIYsense incubated with N-AcMetO, N-AcMet, or BSA, or the inactive form of RIYsense incubated with N-AcMetO. The R/R0 value represents the ratio of the RFI at t = 0 to the current time.

RIYsense biosensor protein underwent in vitro characterization to determine substrate specificity. To examine specificity, 500 µM N-AcMetO and 500 µM N-AcMet were used as substrates. The RFI of N-AcMetO was ~2.4, while the RFI of N-AcMet and no substrate used as a control was ~1.4 (Figure 2A). This demonstrates that only the oxidized form of the Met could induce an increase in the fluorescence intensity of RIYsense. Next, the active and inactive forms of RIYsense were incubated with or without 500 µM N-AcMetO across a pH range of 6.0 to 9.0 (Figure 2B,C). The active form of RIYsense showed an increase in RFI with rising pH, along with a noticeable difference in RFI between incubation with 500 µM N-AcMetO (oxidized) and without it (reduced). In contrast, the inactive form also exhibited an increase in RFI as the pH increased, but no difference was observed between incubation with or without 500 µM N-AcMetO. Next, various concentrations of N-AcMetO (0, 120, 250, or 500 µM) were incubated with a RIYsense biosensor protein for 900 s (Figure 2D). The R/R0 value, which represents the ratio of the RFI at t = 0 to the current time, was recorded every 30 s. The result showed that the R/R0 value increased with a higher concentration of N-AcMetO over time; however, there was no change without N-AcMetO. Then, the R/R0 value was also recorded for 900 s when the active form was incubated with 500 µM N-AcMetO, 500 µM N-AcMet, or 50 µM bovine serum albumin (BSA), or the inactive form was incubated with 500 µM N-AcMetO (Figure 2E). This result showed that only the active form subject to 500 µM N-AcMetO has increased R/R0 value over time, but no changes in others. Consequently, this result shows that RIYsense is specific for the N-AcMetO substrate, mimicking protein-MetO, while it did not respond to protein methionine, as found in N-AcMet and BSA.

### 3.3. High-Throughput Screening to Select MsrB1 Inhibitors by Using RIYsense Biosensor Protein

**Figure 3:**
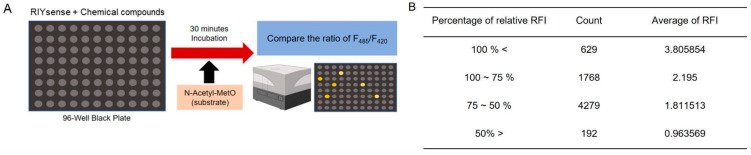
High-throughput screening using RIYsense biosensor protein. (**A**) Illustration of high-throughput screening using RIYsense to identify MsrB1 inhibitors. (**B**) Percentage of RFI decrease obtained by incubating the compounds with RIYsense.

RIYsense is the MsrB1 enzyme-containing biosensor protein that functions based on the specific interaction of the enzyme and its substrate, followed by rapid change in fluorescence. By using this property, 6868 compounds were examined to select inhibitors on 96 wells through high-throughput screening. For this screening, RIYsense was incubated with N-AcMetO as a substrate and a compound as a potential inhibitor, and the RFI value (485 nm/420 nm) was monitored (Figure 3A). The RFI of the oxidized biosensor protein, produced by incubating the biosensor protein with 500 µM N-AcMetO, was 2.706. The RFI of the biosensor protein incubated with both 500 µM N-AcMetO, and a compound was then divided by this value, shown as a percentage of relative RFI. Among the 6868 compounds, 192 were less than 50% in the percentage of relative RFI, which presents 0.964 of the average RFI value (Figure 3B). We selected these compounds as potential MsrB1 inhibitors based on their relatively strong ability to reduce RIYsense fluorescence intensity. They were used for further investigation to confirm their inhibitory function.

### 3.4. Inhibitor Selection by Using Molecular Docking Simulation, NADPH Consumption Assay, and MST Binding Assay

**Figure 4:**
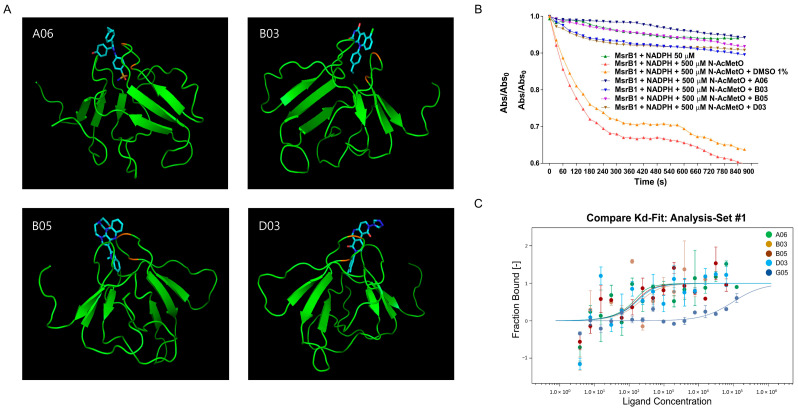
Verification of MsrB1 inhibitors. (**A**) Docking simulation using AutoDock Vina and (**B**) NADPH consumption assay to verify MsrB1 inhibitors, A06, B03, B05, and D03. (**C**) MST binding assay to measure the binding affinity of MsrB1 to A06, B03, B05, D03, and G05.

The 192 compounds identified as MsrB1 inhibitor candidates were further investigated using the AutoDock Vina 1.5.6 software that simulates molecular docking to predict and validate the binding affinity of MsrB1 and inhibitor compounds [35,36]. The grid box was set up at the center of the active site containing catalytic and resolving cysteines, and then docking energy and root mean square deviation (RMSD) were calculated. As a result, among 192 compounds, 37 compounds had negative docking energy and RMSD ≤ 3° (Figure 4A, Table 1) and were selected as potential MsrB1 inhibitors for further investigation. Next, these 37 compounds were evaluated using NADPH consumption assay. In this assay, MsrB1, thioredoxin, thioredoxin reductase, and NADPH were co-incubated with each compound, and the consumption of NADPH was monitored at 340 nm for 900 s. Four compounds (A06, B03, B05, and D03) significantly reduced the NADPH consumption rate, leading to a higher ratio of absorbance at 15 min (900 s) compared to the initial absorbance at 0 min, indicating stronger inhibitory effects than the other compounds. The absorbance ratios were 0.942 for A06. 0.918 for B03, 0.908 for B05, and 0.906 for D03, with the DMSO control showing a ratio of 0.638 (Figure 4B, Table 1). These four compounds were then subjected to a microscale thermophoresis (MST) binding assay to confirm the inhibitory interaction by measuring the affinity between the MsrB1 enzyme and the compounds. Consistently, KD value of A06, B03, B05, and D03 were 28.28 ± 1.21 mM, 97.12 ± 1.60 nM, 47.96 ± 1.14 nM, and 58.18 ± 1.33 nM, respectively, while KD value of the negative control G05 that has no influence on enzyme-substrate interaction was 96,893.81 ± 2.17 nM, indicating that those compounds had a binding affinity to the MsrB1 enzyme for inhibition (Figure 4C, Table 1). As a result, through high-throughput screening using a biosensor protein, 192 compounds were initially identified as inhibitors. Further validation experiments narrowed these down to four compounds, which demonstrated relatively strong inhibitory effects and were found to have high binding affinity to the MsrB1 enzyme.

### 3.5. Biological Applications of MsrB1 Inhibitors

**Figure 5:**
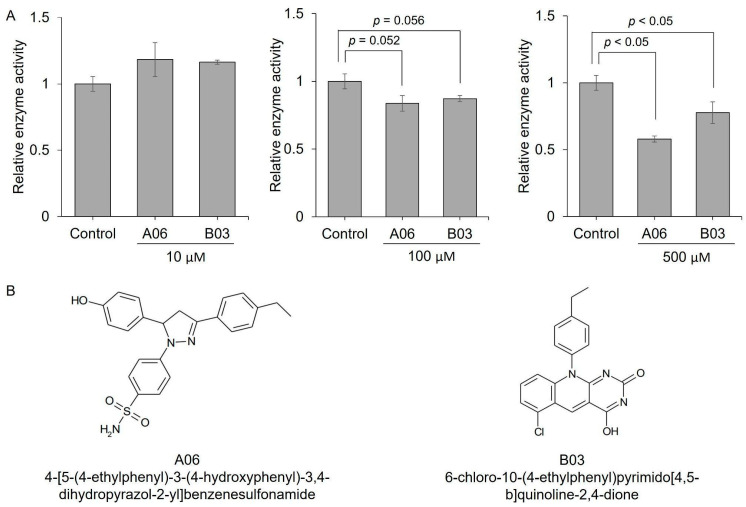
MsrB1 activity assay with various concentrations of MsrB1 inhibitors. (**A**) Relative enzyme activity of MsrB1 was analyzed at 10 µM, 100 µM, and 500 µM MsrB1 inhibitor concentrations. (**B**) Compound structure of A06 and B03 were used in this assay as MsrB1 inhibitors. A06; 4-[5-(4-ethylphenyl)-3-(4-hydroxyphenyl)-3,4-dihydropyrazol-2-yl]benzenesulfonamide and B03; 6-chloro-10-(4-ethylphenyl)pyrimido[4,5-b]quinoline-2,4-dione. All experiments were performed three times.

The catalytic activity of methionine sulfoxide reductases, including MsrB1, can be assessed either indirectly by measuring NADPH consumption or directly by quantifying the conversion of dabsylated methionine sulfoxide to dabsylated methionine [23,37]. The inhibitory effects of compounds A06 and B03, which demonstrated greater inhibition in the NADPH consumption assay compared to other compounds, were evaluated by measuring the direct conversion of methionine sulfoxide to methionine at various concentrations. When each compound was tested at 10 µM in co-incubation with MsrB1 and its substrate, no inhibition was observed. However, at concentrations of 100 µM and 500 µM, relative enzyme activity decreased in a concentration-dependent manner for A06 and B03 (Figure 5). As a result, we confirmed that A06 and B03 act as MsrB1 inhibitors, as demonstrated through various in vitro experiments.

**Figure 6:**
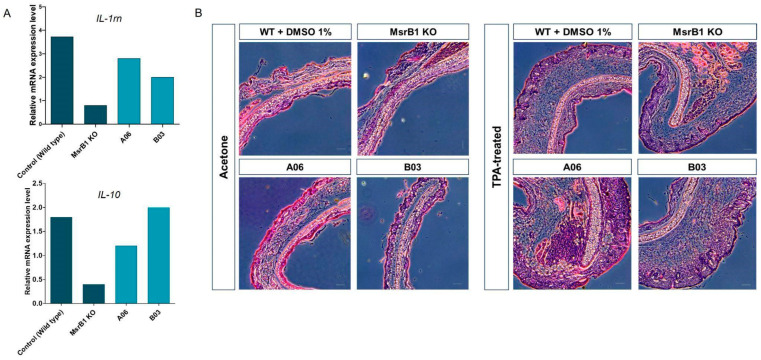
Regulation of the inflammatory response by MsrB1 inhibitors. (**A**) mRNA expression levels of the anti-inflammatory cytokine genes *IL-10* and *IL-1rn* in WT BMDMs treated with MsrB1 inhibitors and in MsrB1 KO BMDMs following LPS stimulation. (**B**) H&E staining was used to visualize the features of acetone-treated left auricles and TPA-treated right auricles of MsrB1 KO and WT mice administered 50 mL water containing 0.5 µM inhibitors or DMSO. The scale bar represents 100 μm. All experiments were repeated two times, and the average values are presented.

Next, it was reported that MsrB1 regulated the expression of anti-inflammatory cytokines such as *IL-10* and *IL-1rn* in bone marrow-derived macrophages (BMDMs) [34,38]. We compared the mRNA expression level of *IL-10* and *IL-1rn* in wild-type BMDMs treated with A06, B03, B05, and D03, as well as in MsrB1 KO BMDMs, following lipopolysaccharide (LPS) stimulation (Figure 6A). Interestingly, *IL-10* expression was significantly reduced in BMDM treated with A06 or B03, while *IL-1rn* expression was reduced in BMDM treated with A06, B03, or B05. This suggests that the regulation of anti-inflammatory cytokine expression in BMDMs might be linked to changes in the catalytic activity of MsrB1, indicating that protein methionine oxidation might play a role in the regulation of this signaling pathway.

As the next step, skin inflammation was examined to assess the inflammatory effects, particularly in chemically irritated skin. Since *IL-10* and *IL-1rn* are key anti-inflammatory cytokines that regulate tissue inflammation, an acute dermatitis model was employed. In this model, the left auricle of wild-type mice treated with A06, B03, and B05 via oral administration, as well as MsrB1 knockout mice, was topically treated with skin irritant 12-O-tetradecanoylphorbol-13-acetate (TPA), while the right auricle was treated with acetone as a vehicle control (Figure 6B). As expected, TPA treatment led to auricular skin swelling and increased thickness, compared to acetone treatment in all samples. Notably, it was observed that the dermal area was substantially more enlarged in both MsrB1 KO and wild-type mice administered with A06, B03, or B05, compared to the wild-type control mice when topically treated with TPA. Consequently, this result proves that A06, B03, and B05, identified as MsrB1 inhibitors, suppress the expression of *IL-10* and *IL-1rn* and, thereby, promote skin inflammation. This is consistent with the previous report that the MsrB1 KO mouse fails to limit inflammatory response due to the lack of anti-inflammatory cytokine expression [34].

## 4. Discussion

MsrB1, a selenoprotein, is distinguished by the presence of selenocysteine in its catalytic site, replacing the more common cysteine residue found in other Msr (methionine sulfoxide reductase) proteins. Throughout evolutionary history, both selenocysteine and cysteine have been alternately incorporated into the catalytic residues of Msr proteins, including both MsrA and MsrB. The presence of selenocysteine in MsrB, particularly in the catalytic site, significantly enhances its enzymatic activity compared to its cysteine-containing counterparts, increasing it by several hundred times [39]. Bacteria and eukaryotes indeed possess different mechanisms and machinery for selenoprotein expression, specifically in how they incorporate selenocysteine, the 21st amino acid. In bacteria, selenocysteine is incorporated into proteins at an in-frame TGA codon, which is traditionally a stop codon. This incorporation occurs when the SECIS (selenocysteine insertion sequence) element is positioned immediately downstream of this TGA codon within the same operon. The proximity of the SECIS element to the TGA codon in bacteria allows for efficient recognition and incorporation of selenocysteine by the bacterial translational machinery. In contrast, eukaryotes, including mammals, also use an in-frame TGA codon to encode selenocysteine, but the SECIS element is located in the 3′ untranslated region (3′ UTR) of the mRNA, far from the codon itself. This spatial separation requires a different set of molecular components and additional factors for successful selenocysteine incorporation. Because the SECIS element in eukaryotes is in the 3′ UTR, the translation machinery must interact with this distant element to recruit the necessary factors for incorporating selenocysteine at the TGA codon. Due to these fundamental differences in selenoprotein expression systems, mammalian MsrB1, which relies on the eukaryotic mechanism of selenocysteine incorporation, cannot be properly expressed in *E. coli*. The bacterial system lacks the necessary machinery to recognize and process the eukaryotic SECIS element located in the 3′ UTR, leading to the inability to express and purify functionally active recombinant mammalian MsrB1 in *E. coli* for enzymatic studies. This presents a significant challenge for researchers attempting to study the enzymatic properties of MsrB1 using bacterial expression systems. An alternative approach for studying MsrB1 enzymatic activity involves using a version of MsrB1 in which the selenocysteine is replaced by cysteine. This cysteine-containing variant of MsrB1 can be expressed in *E. coli* and used for enzymatic studies. However, this substitution presents a significant challenge because cysteine-containing MsrB1 exhibits substantially reduced catalytic activity compared to its selenocysteine-containing counterpart [39]. The reduced activity makes it less ideal for certain applications, such as inhibitor screening, where higher enzyme activity is often required to detect subtle changes in enzyme inhibition. Despite this limitation, the RIYsense biosensor protein employs a cysteine-containing MsrB1 for its function. The RIYsense biosensor protein is designed with a MsrB1, cpYFP, and a thioredoxin domain in close proximity within a single-polypeptide chain. This unique configuration allows the biosensor protein to respond sensitively and immediately to substrate binding. The proximity of the thioredoxin and MsrB1 enables efficient interaction and rapid conformational changes in cpYFP upon substrate interaction, compensating for the lower catalytic activity of the cysteine-containing MsrB1. This design allows for a convenient and effective way to monitor substrate binding and enzyme activity, providing valuable insights even when using the less active cysteine variant of MsrB1 for inhibitor screening.

Up-to-date, much evidence underscores the importance of MsrB1 in pathophysiological regulation. Initially characterized as an antioxidant enzyme that protects against oxidative stress by reducing methionine sulfoxide residues in proteins, MsrB1 is now recognized for its broader role in cellular physiology. This expanded understanding suggests that MsrB1’s function may go beyond simple antioxidant defense; it might be involved in the redox regulation of substrate proteins, influencing various cellular processes. This redox regulation could impact protein function, signaling pathways, and cellular responses, highlighting MsrB1’s potential significance in health and disease beyond its traditional role in mitigating oxidative damage [40]. Indeed, MsrB1 has been shown to be associated with certain pathological conditions, including the incidence and development of several cancers, modulation of the immune response, regulation of brain function, aging, etc. [40,41,42]. Furthermore, the function of certain proteins, including actin, CamKII, and β-amyloid peptide, was controlled by MsrB1 via the reversible oxidation and reduction in specific methionine residues [43,44]. Accordingly, MsrB1 inhibitors could serve as powerful tools for studying the pathological conditions and molecular mechanisms regulated by MsrB1.

## 5. Conclusions

Approximately 6800 compounds from the representative library deposited by the Korea Chemical Bank were obtained for MsrB1 inhibitor screening. This library is a primary screening collection representing a total of 640,000 compounds held by the Korea Chemical Bank, selected for diversity in their chemical structures and representativeness. These compounds have undergone drug-likeness selection processes, such as the “Rule of 5”, and their molecular weight and purity have been verified through LC/MS. Using this library, 192 compounds showing an inhibitory effect of more than 50% were initially selected via the RIYsense biosensor protein. However, since certain compounds may exhibit fluorescence characteristics, induce pH changes, or inhibit trx, making it difficult to accurately measure the inhibition effect, a second round of selection was conducted using molecular docking simulation. This simulation identified compounds likely to act as competitive inhibitors by binding to the MsrB1 enzyme region of the RIYsense biosensor protein and inhibiting substrate binding. Next, an in vitro assay using the NADPH consumption assay was conducted to confirm whether these compounds exhibited actual inhibitory effects. Four compounds with the highest inhibitory effects were selected, and additional MST binding assays confirmed their binding affinity to the MsrB1 enzyme. Subsequently, another in vitro assay, the HPLC assay using dabsylated methionine sulfoxide, was performed to assess MsrB1 activity. Two compounds were finally selected for their concentration-dependent inhibitory effects. The NADPH consumption assay measures the oxidation of NADPH, but since NADPH naturally oxidizes over time and oxidation can also be accelerated by the compounds themselves, the HPLC assay, which directly measures the reduced substrate, was used for final validation. Although it cannot be ruled out that some of the compounds excluded in earlier stages may still exhibit inhibitory effects, two compounds demonstrating the most definitive inhibitory effects were ultimately selected and tested for their potential to control inflammation. In summary, the multi-step screening process, thorough in vitro validation, and diverse methods of measuring enzyme activity make this study one of the more rigorous and comprehensive investigations of enzyme inhibitors. While similar papers may employ some of these techniques, the combination of all these approaches sets this study apart in terms of precision, speed, and reliability. This process not only enhances the accuracy of identifying true MsrB1 inhibitors but also helps prevent time loss in later stages of drug development, effectively offsetting the initial time investment during the screening and validation stages.

## Figures and Tables

**Table 1:** Data from molecular docking simulations, NADPH consumption assays, and MST binding assays for chemical compounds targeting MsrB1.

	Molecular Docking Simulation				NADPH Consumption Assay	MST Binding Assay
	Mode	Affinity (Kcal/mol)	RMSD L.B. (Å)	RMSD U.B. (Å)	NADPH (Abs_15min_/Abs_0min_)	K_D_ ± SD (nM)
A06	5	−6.5	1.997	3.012	0.942	28.28 ± 1.21
B03	3	−6.4	2.407	4.36	0.918	97.12 ± 1.60
B05	2	−4.5	1.036	1.757	0.908	47.96 ± 1.14
D03	2	−7.4	0.799	1.412	0.906	58.18 ± 1.33
G05	5	−7.5	1.990	2.983	0.657	96,893.81 ± 2.17

## Data Availability

Data are contained within the article.
